# Relationship between Ultra-Processed Food Consumption and Risk of Diabetes Mellitus: A Mini-Review

**DOI:** 10.3390/nu14122366

**Published:** 2022-06-07

**Authors:** Muneerh I. Almarshad, Raya Algonaiman, Hend F. Alharbi, Mona S. Almujaydil, Hassan Barakat

**Affiliations:** 1 Department of Food Science and Human Nutrition, College of Agriculture and Veterinary Medicine, Qassim University, Buraydah 51452, Saudi Arabia; 411200219@qu.edu.sa (M.I.A.); 411200162@qu.edu.sa (R.A.); hf.alharbi@qu.edu.sa (H.F.A.); m.almujaydil@qu.edu.sa (M.S.A.); 2 Department of Food Technology, Faculty of Agriculture, Benha University, Moshtohor 13736, Egypt

**Keywords:** ultra-processed food, type 2 diabetes, gestational diabetes

## Abstract

Studying the factors that cause diabetes and conducting clinical trials has become a priority, particularly raising awareness of the dangers of the disease and how to overcome it. Diet habits are one of the most important risks that must be understood and carefully applied to reduce the risk of diabetes. Nowadays, consuming enough home-cooked food has become a challenge, particularly with modern life performance, pushing people to use processed foods. Ultra-processed food (UPF) consumption has grown dramatically over the last few decades worldwide. This growth is accompanied by the increasing prevalence of non-communicable diseases (NCDs) such as cardiovascular diseases, hypertension, and type 2 diabetes. UPFs represent three main health concerns: (i) they are generally high in non-nutritive compounds such as sugars, sodium, and *trans* fat and low in nutritional compounds such as proteins and fibers, (ii) they contain different types of additives that may cause severe health issues, and (iii) they are presented in packages made of synthetic materials that may also cause undesirable health side-effects. The association between the consumption of UPF and the risk of developing diabetes was discussed in this review. The high consumption of UPF, almost more than 10% of the diet proportion, could increase the risk of developing type 2 diabetes in adult individuals. In addition, UPF may slightly increase the risk of developing gestational diabetes. Further efforts are needed to confirm this association; studies such as randomized clinical trials and prospective cohorts in different populations and settings are highly recommended. Moreover, massive improvement in foods’ dietary guidelines to increase the awareness of UPF and their health concerns is highly recommended.

## 1. Introduction

In recent decades, dramatic growth in the consumption of ultra-processed foods (UPFs) has been reported worldwide. The United States (US) and the United Kingdom (UK) reported the highest consumption ratios, accounting for more than half of the total calories consumed. In contrast, Italy reported the lowest, which accounted for almost 10% of the total calories consumed. Other regions, such as Australia, had an average of 40%. Asian countries such as Korea, Japan, Malaysia, and Indonesia had an average of 25.8%, 28.2%, 29%, and 19.5%, respectively, and for Middle Eastern countries, a Lebanese study reported an average of 36.5% of the total calories consumed [1]. UPFs are becoming more present in all food categories, even in infants’, children’s, and adolescents’ diets [2]. This global phenomenon is accompanied by an increase in the prevalence of overweight/obesity, metabolic syndrome, and other non-communicable diseases (NCDs) such as cardiovascular diseases, hypertension, and type 2 diabetes (T2D) [3]. More than 500 million adults aged 20–79 years live with diabetes worldwide; by 2030, this number will rise to more than 600 million and more than 700 million by 2045. Indeed, more than 6 million deaths occurred because of diabetes in 2021. Diabetes also resulted in at least 966 billion US dollars in health expenditure [4].

UPFs are products that have undergone a series of industrial processes, including physical, biological, or chemical processes, coupled with the use of additives such as colorings, emulsifiers, and preservatives [5,6,7]. A privilege of this industry is providing hyper-palatable products that are easy to advertise and can last on store shelves or kitchen cabinets as long as possible with the least amount of expense, therefore gaining maximum profits [3]. The rise in the consumption of UPFs, including “fast foods”, “soft and sugary drinks”, “processed meats”, and other types of “ready-to-consume” foods, has been linked to the risk of obesity and many other NCDs such as cardiovascular disease, hypertension, and depression [8]. UPFs are characteristically poor in nutritional compounds and high in non-nutritive components [3,5,9]. In addition, the use of several additives may cause multiple health issues, and the packaging may also contain harmful substances [6]. These health concerns are questionable if they could be associated with the rapid increase in the prevalence of diabetes; the objective of this paper is to discuss the relevant findings related to the association between UPF consumption and the risk of diabetes. Articles published between January 2018 and January 2022 were searched on PubMed, Science Direct, and EBSCO. Further search was conducted on Google Scholar using the following keywords: “ultra-processed foods”, “NOVA”, “type 2 diabetes”, “body mass index” and “diabetes”, “gestational diabetes mellitus”, and “non-communicable diseases”.

## 2. An Overview of Ultra-Processed Foods

Nowadays, almost all available food products are processed; the processing techniques are not the issue. Multiple conventional and other relatively novel processes such as drying, non-alcoholic fermentation, freezing, pasteurization, and vacuum-packing are beneficial for either human health or preserving foods. The issue is with the term “ultra-processed”, which reflects the series of industrial processes that have been undergone [10]. Multiple food classification systems have been designed to characterize foods according to processing criteria. In 2010, Monteiro and colleagues [11] established a system known as “NOVA”. Since then, it has become the most widely used system in the research field [2,6,12].

**Figure 1:**
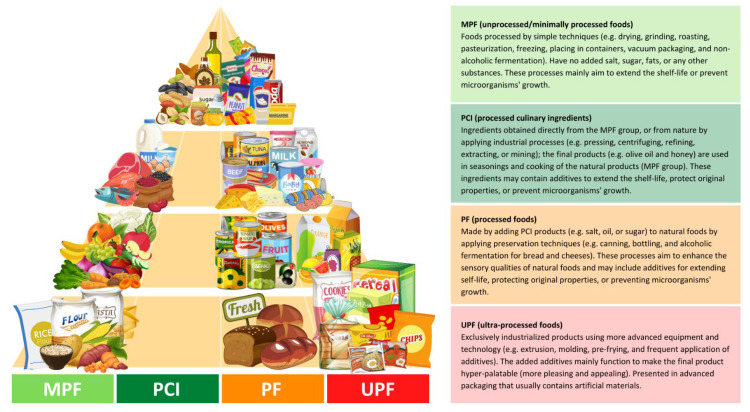
Distributed food examples in the food pyramid based on the NOVA classification, according to the given criteria by Monteiro et al. [13]. The different colors (light green, green, orange, and red) represent the degree of the processing; green colors (MPF and PCI) have the least level of industrial processing, orange (PF) has a modest level, and red (UPF) has the most extensive level.

NOVA classifies foods based on the degree and purpose of industrialized processing into four categories (Figure 1): (i) unprocessed/minimally processed foods, (ii) processed culinary ingredients, (iii) processed foods, and (iv) ultra-processed foods [13]. The latter, ultra-processed foods (UPFs), are products that have been manufactured by different types of industrial processes, including physical, biological, or chemical processes, such as hydrogenation, extrusion, and pre-frying, coupled with the use of “cosmetic additives” such as colorings, flavorings, sweeteners, and emulsifiers [5,6,7]. Cosmetic additives are low-cost ingredients that have rarely or never been used in kitchens. The presence of at least one cosmetic additive in any product’s ingredients list, which by law must be included in food labels, identifies it as ultra-processed [13]. These additives are applied in the food industry to provide long-shelf-life products that are more convenient, hyper-palatable, and affordable to consumers with the least economic costs and maximum profit [3,6].

UPFs are products mainly characterized by poor nutritional value as they are extremely high in non-recommended nutrients (e.g., free sugars, sodium, saturated and *trans* fats) that are linked to the risk of multiple NCDs such as cardiovascular diseases, cancer, and T2D, and are low in dietary fiber, proteins, and potassium. They also tend to contain highly refined grains and low levels of several bioactive components [3,5,9]. In an Australian cross-sectional study [14], UPF products were found to contain 4.7, 2.9, and 1.9 times more free sugars, sodium, and energy density, respectively, and 1.7 and 1.4 times less potassium and fiber, respectively, than non-ultra-processed products. Increased intake of these non-recommended nutrients was in a statistically significant linear relationship (*p* < 0.001) with the increase in UPF consumption. From the lowest UPF consumption ratio to the highest, the proportions of free sugars, *trans* fats, and energy density increased remarkably from 22% to 82%, 6% to 11%, and 2% to 25%, respectively.

Consistently, participants who consumed the highest level of UPF (contributing to an average of 74.5% of total energy intake (TEI)) had exceeded their upper limits of recommended intakes of free sugars, saturated fats, and sodium compared to those who consumed the lowest levels (average UPF, 12.8% of TEI). In addition, over 85% of them did not meet their recommended intakes of dietary fiber and potassium. Similarly, a British cross-sectional [15] study also found a significant relationship between increased intake of UPFs and an increase in carbohydrates, free sugars, total fats, saturated fats, and sodium intake, while the intake of proteins, fibers, and potassium showed a decrease. From the least UPF consumption ratio up to the highest, total energy intake derived from free sugar intake increased from 9.9% to 15.4%, and that derived from dietary fiber intake decreased from 8.36% to 6.86%. A positive association between UPF consumption and increased intake of non-recommended nutrients has also been reported in other studies [16,17,18].

Beyond the poor nutritional value, the cosmetic additives used in this industry may cause multiple health issues. Some additives such as monosodium glutamate (a flavor enhancer), carrageenan (a thinking agent), soya lecithin (an emulsifier), and sucralose or aspartame (non-nutritive sweeteners) are widely used in the food industry [2,6,13]. However, they might be associated with multiple health risks, including diabetes. Consumption of monosodium glutamate, for example, has been suggested as a potential factor in the development of obesity and diabetes, as well as other side effects such as hepatotoxic, neurotoxic, and genotoxic effects [19]. Monosodium glutamate may increase oxidative stress due to elevated lipid peroxidation; a dose of 4 g kg^−1^ of body weight was orally administrated to mice for only seven days and significantly showed an increase in lipid peroxidation [20]. Similar results were found in a recent investigation; after eight weeks of administration, a dose of 2.5 g kg^−1^ of body weight significantly caused oxidative stress in rat modules [21]. Another study showed that a dose of 0.75 g/kgof body weight could induce diabetes in rat modules due to elevated fasting blood glucose levels [22]. Carrageenan might also be associated with the development of diabetes; it was shown in an in vivo and in vitro investigation that exposure to carrageenan led to an impairment in glucose intolerance and a rise in both insulin resistance and insulin signaling [23]. Sucralose was also associated with elevating glucose and insulin levels and slowing down the clearance of insulin from the plasma of obese individuals [24].

Furthermore, the packaging of UPFs doubles these concerns, as they are made of different kinds of synthetic chemicals, and their safety has been questioned recently. Chemicals such as bisphenols and phthalates are commonly used in food plastic packaging [6,25]. An analog of bisphenols, bisphenol A (BPA), has been banned in many countries due to its harmful effects on multiple health issues such as cardiometabolic disorders and cancer. However, it has been replaced by another bisphenol analog, bisphenol S (BPS), indicated as a “regrettable substitution”. Many researchers have pointed out that restrictions placed on BPA must be placed on BPS as well. BPS is suspected to be absorbed orally much more than BPA. In addition, multiple studies have correlated exposure to BPS with numerous health risks, including diabetes [26]. Like bisphenols, exposure to phthalates has been associated with various health effects, including obesity and insulin resistance. It was shown that UPF consumption was correlated with increased exposure to phthalates [6,25,27].

## 3. Ultra-Processed Foods and Health Outcomes

Concerns surrounding UPFs have attracted researchers’ interest all over the world; several studies in different populations and settings have found an association between elevated intake of UPFs and all-cause mortality and many other different specific health outcomes such as cardiovascular disease, hypertension, overall cancer, depression, metabolic syndrome, overweight and obesity, and gestational obesity [5]. The risk of all-cause mortality has been reported to increase by 62% with the high UPF consumption; with each additional serving of UPF products, the risk increased by 18% [17]. An increased risk of cardiovascular disease by 11% [28] and overall cancer by 10% [29] has been reported with each 10% increase in UPF intake. The results remained significant even after further adjustment of several nutritional quality markers. Among adult individuals followed up for a median of 10.3 years, higher UPF consumption was reported to increase the risk of developing depression by 31% [30]. The risk of developing metabolic syndrome has significantly increased by 28%, with high UPF consumption contributing to an average of ≥71% of TEI [16]. Results showed a statistically significant association between high UPF consumption, elevated waist circumference, and reduced high-density lipoprotein (HDL) cholesterol levels. An increase in the risk of obesity by 31% was reported, with an average UPF consumption contributing to 73% of TEI [31]. A recent systematic review reported that high UPF consumption correlates linearly with elevated body mass index (BMI) [8]. Several studies had reported similar significant results [17,18,27]; elevated BMI levels were observed among individuals consuming a high level of UPF. Obesity, as well established for decades, is a major leading cause of the development of T2D [32]. Indeed, higher BMI levels above 29 kg/m^2^ were linked to the development of T2D by 10 times more than normal levels [32]. Higher UPF consumption can also increase fasting blood glucose, insulin, and the homeostatic model assessment for insulin resistance (Homa-IR). A study comparing the effect of unprocessed and UPF diets on obesity has reported that the unprocessed diet resulted in a reduction in levels of the three parameters; such desirable results were not observed after the consumption of the UPF diet [33].

## 4. Ultra-Processed Foods and Risk of Diabetes

Regardless of the strong evidence between high consumption of UPF and obesity that has been mentioned in the above section, multiple investigational studies have assessed the association between UPF and the incidence of diabetes, especially T2D, and a few other studies investigated the association with gestational diabetes (Table 1).

The Canadian cross-sectional study by Nardocci and colleagues [31], based on the 2015 Canadian Community Health Survey–Nutrition data, showed a strong association between high UPF consumption and obesity and reported a significant association with developing diabetes. Those who consumed a high level of UPF (average UPF intake, 73% of TEI) had an increased risk of developing diabetes by 37% compared to those who consumed the lowest level of UPF (average UPF, 24% of TEI). With each 10% increase in energy intake derived from UPF intake, there was an increase in both obesity and diabetes risk by 6%. These findings align with a large French cohort [34] where adult individuals were followed up for a median of six years, and UPF consumption was measured based on grams percentage (in weight). The higher UPF consumption (average proportion (in weight) in the diet was 17.29%) showed a significant linear relationship with developing T2D. Results remained statistically significant even after further adjustment of multiple covariates, including the nutritional quality of the diet. The nutrient intake profile was also similar to the previously mentioned studies; the high UPF consumption was significantly associated with a higher intake of energy, saturated fats, sodium, and sugars and a lower fiber intake. Another cohort carried out in the UK [35] also revealed a strong relationship between high consumption of UPF and the development of T2D. The findings showed that for every 10% increase in UPF consumption, the incidence of T2D increased by 12%. More remarkably, over 5.4 years of follow-up, individuals who consumed the highest levels of UPF (average proportion (in weight) in the diet was 41.9%) had an increased risk of developing T2D by 44% compared to those who consumed the lowest levels of UPF (average UPF proportion was 7.7%). A recent cohort carried out in Spain [36] also found that individuals who consumed a high level of UPF had an increased risk of developing T2D by 53% relatively compared to those who consumed a lower level of UPF. This relationship was in a significant dose-dependent manner (*p* = 0.024). Higher BMI levels were also observed in those who consumed a high level of UPF. Surprisingly, the mean UPF proportion (in weight) in the diet was 9.5%, which is less than that found in both the French [34] and the British [35] cohorts, yet showed a significant association with the risk of T2D. On the other hand, the nutritional intake of those who consumed the highest levels of UPF had elevated levels of fats and decreased levels of proteins.

From another point of view, and due to similarities between the results of the British [35] and the French [34] cohorts, it was indicated that increased risk of T2D might be associated with all kinds/categories of UPF products (e.g., beverages, sugary products, processed meats, or processed bakery products) [35]. The differences in types of UPF consumed between these two cohorts are the main reason; the consumed UPF products in the French cohort were mainly sugar-based, ultra-processed fruits and vegetables and beverages, which represented 28%, 18%, and 16%, respectively, of total UPF consumption. In comparison, the most-consumed UPF products in the British cohort were beverages, industrial-processed bakery products, breakfast cereals, and industrial-processed frozen/shelf-stable prepared meals, which represented 39.1%, 29.9%, and 19.3%, respectively, of total UPF consumption. Hence, it is likely that the type/category of UPF consumed does not alter the association with the increased risk of T2D. However, another large cohort carried out in the Netherlands earlier this year [37] showed that the types of UPFs were significantly altering the results. However, in this study, the difference was between savory snacks, high in salt and sodium content, and traditional Dutch cuisine. The savory snacks showed a significant positive relationship with the incidence of T2D, while the traditional Dutch cuisine had no relation. According to the authors, sliced bread was the main product consumed in traditional Dutch cuisine, and because the majority of sliced bread consumed in the Netherlands is brown bread, which is made of a combination of whole-wheat and white flour or whole-wheat flour only, it is higher in fiber and micronutrients. High fiber intake is beneficial for preventing and managing T2D [42,43]. Therefore, nutritional quality is a key factor in altering the effect of UPF on developing T2D. The Dutch participants who consumed the highest levels of UPF (mean UPF proportion (in weight) in the diet was 48.7%) had a remarkably increased risk of developing T2D by 80% compared to those who consumed the lowest level of UPF (mean UPF proportion was 23.7%). It is worth mentioning that for each 10% increase in UPF consumption, the risk increased by 25%.

Consumption of sweet drinks and salty processed foods was found to enormously increase the risk of prediabetes by 248% and 48%, respectively, and the risk of T2D by 219% and 600%, respectively, compared to individuals who did not consume these products or had a rare consumption ratio. In contrast, the consumption of at least three servings or more each of fruit and vegetables a day decreased the risk of prediabetes by 26% and 5%, respectively, and the risk of developing T2D by 12% and 5%, respectively, compared to those who did not [44]. Consistently, consumption of an unprocessed diet among diabetic outpatients led to a significant reduction in fasting plasma glucose, glycosylated hemoglobin (HbA_1c_), and low-density lipoprotein (LDL) cholesterol compared to patients who consumed an unhealthy diet that mostly consisted of high levels of sweets and desserts, refined carbohydrates, and UPFs [45]. It was indicated that reduced LDL cholesterol levels are highly related to consuming an unprocessed diet. The administration of cholesterol-lowering drugs was not different among these participants, and thus, the results could not be related to drug usage. In addition, the patients who consumed the non-healthy diet had a nutritional profile significantly higher in saturated fats, which has been linked for decades with elevated LDL levels [46]. Adherence to dietary recommendations was shown in a French cohort to be significantly related to the development of T2D [47]. Participants were followed up for a median of 6.7 years, and their adherence to dietary guidelines of a French nutrition and health program, Programme National Nutrition Santé (PNNS), was assessed. After adjusting several covariates, including BMI, those who had high adherence to these guidelines had a significantly decreased risk of developing T2D by 49% compared to those who had the lowest adherence ratio, indicating that following the nutritional recommendations is strongly related inversely to developing T2D.

Another matter of interest, high consumption of UPF, might also be associated with developing gestational diabetes; a cross-sectional study [38] showed that UPF consumption strongly increases gestational weight but with no association with gestational diabetes. However, the classification of foods based on the degree of industrial processing was not fully achieved in this study. Therefore, misclassification may alter or attenuate the association between UPF and gestational diabetes. In contrast, a recent cohort [39] found that high UPF consumption was significantly associated with developing gestational diabetes (*p* = 0.041); however, this association was not observed among women under 30 years old. This could be explained by changes in the immune system that are reported with aging [48,49]. Cytokine dysregulation is one of the remarkable changes that lead to a chronic low-grade inflammatory state due to elevated pro-inflammatory cytokines, thus increasing the risk of comorbidities, including insulin resistance. Furthermore, another cohort that investigated the effect of UPF consumption on glycemic control among pregnant women with pre-gestational diabetes [40] showed that in the third trimester, each kcal of energy intake derived from UPF consumption significantly increased the one-hour postprandial glucose level, HbA_1c_, and gestational weight by 0.143 mg/dL, 0.007%, and 0.11 kg, respectively. Yet, the overall results showed that high UPF consumption is mostly associated with gestational weight gain.

## 5. Future Approach

The findings of the presented studies showed a strong association between UPF consumption and the incidence of diabetes. Almost all of these studies had adjusted their results for various covariates, which increased the significance of the findings. However, these studies were mainly conducted in Europe and America; other studies conducted in regions such as East Asia and/or the Middle East or other different regions are lacking. Recent investigations show that genetic differences between European and Asian populations, for example, play a significant role in the pathogenesis of diabetes [50]. Therefore, further studies in different populations and settings are highly needed to confirm the association. In addition, studies related to UPF intake and gestational diabetes are lacking worldwide. More studies on this particular point are also required.

## 6. Conclusions

The relevant findings showed that UPF consumption is significantly associated with the incidence of obesity, increasing insulin resistance, BMI levels, waist circumference, and LDL cholesterol levels while reducing HDL cholesterol levels, leading to a potential increase in the risk of T2D. Multiple investigational studies have revealed an association between the consumption of UPF and the risk of T2D, where the mean contribution of UPF proportion (in weight) in the diet ranged from 9.5% to 48.7%. In addition, there might be a slight association between high UPF consumption and gestational diabetes. These findings could be related to the significant and remarkable increase in the non-recommended nutrients found with high UPF consumption. Although this is not the only concern, additives included in this industry may cause health issues that have not been adequately investigated. Additionally, the packaging of UPFs may contain harmful chemicals, which have also not been well studied.

Further studies, such as randomized clinical trials and prospective cohorts in different populations and settings, are needed to confirm these findings and take urgent actions to improve future dietary guidelines to emphasize the detrimental effects of UPF to limit their consumption as much as possible.

## Figures and Tables

**Table 1:** Summary of studies investigating the association between ultra-processed food consumption and risk of diabetes and gestational diabetes.

Study Design/Follow-Up/	UPF Intake Assessment	Main Results	References
Participant Characteristics			
Cross-sectional/- *n* = 13,608 adults (age ≥ 19 years) Diabetic (7%) (50% women)	24 h recalls/NOVA/ proportion of TEI	↑ DM risk by 37% (with high vs. low intake, 73% vs. 24% of TEI) An absolute 10% increase in UPF intake increases the risk by 6% (*p* < 0.05)	Nardocci et al. [31] (2021, Canada)
Prospective cohort/6 years *n* = 104,707 adults (age ≥ 18 years) Non-diabetic (79.2% women)	24 h recalls/NOVA/ proportion of weight	An absolute 10% increase in UPF intake was associated with 15% higher risk of T2D (*p* = 0.001)	Srour et al. [34] (2020, France)
Prospective cohort/5.4 years *n* = 21,730 adults (age 40–69 years) Non-diabetic (52.9% women)	24 h recalls/NOVA/ proportion of weight	↑ T2D risk by 44% (with high vs. low intake, 41.9% vs. 7.7% of diet proportion) (*p* < 0.028)	Levy et al. [35] (2020, UK)
Prospective cohort/12 years *n* = 20,060 adults (age ≥ 18 years) Non-diabetic (61.5% women)	FFQ/NOVA/ proportion of weight	↑ T2D risk by 53% (with high vs. low intake, >323.3 vs. <214.6 g/day of diet proportion) (*p* = 0.024)	Llavero-Valero et al. [36] (2021, Spain)
Prospective cohort/41 months *n* = 70,421 adults (age 35–70 years) Non-diabetic at baseline (58.6% women)	FFQ/NOVA/ proportion of weight	↑ T2D risk by 80% (with high vs. low intake, 48.7% vs. 23.7% of diet proportion) An absolute 10% increase in UPF intake increases the risk by 25% (*p* < 0.001)	Duan et al. [37] (2020, The Netherlands)
Cross-sectional/- *n =* 785 pregnant women (age ≥ 20 years) Non-diabetic at baseline	24 h recalls/*/ proportion of TEI	↑ gestational obesity risk by 3 times (with high vs. low intake, 47% vs. 18% of TEI) (*p* < 0.05) No association with GDM (*p* > 0.05)	Sartorelli et al. [38] (2019, Brazil)
Prospective cohort/7.2 years *n =* 3730 pregnant women (age 18–49 years) Non-diabetic	FFQ/NOVA/ proportion of weight	↑ GDM risk by 10% (with high vs. low intake, >4.5 vs. <3.3 serving/day)(*p* = 0.818) women aged ≥30 years had a doubled risk (*p* = 0.041)	Leone et al. [39] (2021, Spain)
Cohort/- *n =* 42 pregnant women (age ≥ 20 years) pre-gestational diabetics	FFQ/NOVA/ proportion of TEI	Each 1 kcal from UPF in the 3rd trimester (mean intake, 15.2% of TEI): ↑ 1-h PPG level by 0.143 (*p* = 0.011) ↑ HbA_1c_ by 0.007% (*p* = 0.025) ↑ gestational weight by 0.11 kg (*p* = 0.006)	Silva et al. [40] (2021, Brazil)

Abbreviations: UPF: Ultra-processed foods; FFQ: Food frequently questionnaires; TEI: Total energy intake; DM: Diabetes mellitus; T2D: Type 2 diabetes; GDM: Gestational diabetes mellitus; PPG: Postprandial glucose; HbA1c: glycosylated hemoglobin. * Classified based on the 2014 Guia Alimentar para a População Brasileira; see Louzada et al. [41] for the detailed method, (↑): meaning increased.

## Data Availability

Data is contained within the article.
